# Food Insecurity in the Rural United States: An Examination of Struggles and Coping Mechanisms to Feed a Family among Households with a Low-Income

**DOI:** 10.3390/nu14245250

**Published:** 2022-12-09

**Authors:** Carmen Byker Shanks, Lauri Andress, Annie Hardison-Moody, Stephanie Jilcott Pitts, Megan Patton-Lopez, T. Elaine Prewitt, Virgil Dupuis, Karen Wong, Marisa Kirk-Epstein, Emily Engelhard, Monica Hake, Isabel Osborne, Casey Hoff, Lindsey Haynes-Maslow

**Affiliations:** 1 Gretchen Swanson Center for Nutrition, Omaha, NE 68154, USA; 2 Department of Health and Human Development, Montana State University, Bozeman, MT 58717, USA; 3 Commonwealth School of Medicine, Geisinger Health, Danville, PA 17821, USA; 4 Agriculture and Human Sciences, North Carolina State University, Raleigh, NC 27695, USA; 5 Department of Public Health, East Carolina University, Greenville, NC 27858, USA; 6 Health and Exercise Science, Western Oregon University, Monmouth, OR 97361, USA; 7 Fay W. Boozman College of Public Health, University of Arkansas, Little Rock, AR 72205, USA; 8 Extension, Salish Kootenai College, Pablo, MT 59855, USA; 9 Share Our Strength, Washington, DC 20005, USA; 10 Feeding America, Chicago, IL 60601, USA; 11 Healthy Policy and Management, University of North Carolina, Chapel Hill, NC 27514, USA

**Keywords:** food security, rural, dietary intake, health disparities

## Abstract

Households with a low-income in rural places experience disproportionate levels of food insecurity. Further research is needed about the nuances in strategies that households with a low-income in rural areas apply to support food security nationally. This study aimed to understand the barriers and strategies that households with a low-income in rural areas experience to obtain a meal and support food security in the United States. We conducted a qualitative study with semi-structured interviews among 153 primary grocery shoppers with a low-income residing in rural counties. A majority of family’s ideal meals included animal-based protein, grains, and vegetables. Main themes included struggles to secure food and coping mechanisms. Ten categories included affordability, adequacy, accommodation, appetite, time, food source coordinating, food resource management, reduced quality, rationing for food, and exceptional desperation. These results can inform public health professionals’ efforts when partnering to alleviate food insecurity in rural areas.

## 1. Introduction

According to the United States (U.S.) Department of Agriculture’s Household Food Security Survey Module, food insecurity has persisted over time [1]. For the past 20 years, food insecurity rates have ranged between 10% and 15% [1,2]. Food insecurity is largely driven by poverty across socioeconomic status, demographics, and geographies. Households with a low-income, defined as below 185% of the federal poverty threshold are at the greatest risk for food insecurity [2]. Further, poverty is also stratified by systemic inequities that cause certain racial and ethnic groups to experience negative effects more severely than other groups [3,4,5,6]. For example, food insecurity rates disparately increased among these populations during the COVID-19 pandemic [2,7,8].

Patterned by geography and socioeconomic status, a myriad of research demonstrates households with a low-income in the U.S. that are also food insecure are at even greater risk for no or low access to healthy and affordable food, poor diets, nutritional deficiencies, and worse health outcomes than households with a higher income [9,10]. Evidence shows that healthy diets cost more in money and time while less healthy food, so easily accessible in the U.S., typically costs less in money and time [11,12,13,14,15,16]. Further, healthy food is less accessible in neighborhoods or geographic areas with low-income households, including rural communities [13,17,18,19,20]. Ultimately, less financial and geographic access to healthy foods leads to lower dietary quality among those in rural, households with a low-income.

Findings from dietary patterns observed annually in National Health and Nutrition Examination Survey (NHANES) demonstrate that individuals from households with a low-income are less likely to meet the Dietary Guidelines for Americans (DGA) than households with higher incomes [21,22]. Notably, American eating patterns for the general population have not met the recommendations put forth in the DGA, especially for vegetables, fruit, and dairy, and this gap widens among individuals in households with a low-income [23,24].

Seligman and Berkowitz (2019) review strategies that lower income households broadly utilize to confront food security [25]. The strategies implemented typically begin at the individual and household level (i.e., skipping meals, eating low-quality foods, using coupons to save cost on food), and as resources are depleted, strategies from the community and systems levels are employed (i.e., rely on informal support from family/friends, obtain food from pantry/soup kitchen, enroll in food assistance program) [24]. Yet, the type, timing, and mix of strategies employed by low-income, rural households are underexplored.

When compared to other geographies in the U.S., rural areas have consistently higher poverty rates than urban areas, at 16.1% versus 12.6%, respectively, and greater food insecurity rates than the overall population, at 12.1% in rural communities versus 10.5% of all households, respectively [26,27]. Economic instability in rural regions, as evidenced by job loss, declining populations, and growing elderly populations, creates low food access, transportation barriers, and financial insecurity [28,29]. On the other hand, rural communities possess positive assets that mitigate rural economic instability and contribute to building food security including social connectedness, access to land and agriculture, and the presence of local businesses [29,30,31,32,33]. Additionally, enrollment in federal nutrition assistance programs has shown to be higher in some rural versus urban areas [34,35].

This research explores the experiences that households with a low-income in rural areas face to obtain a meal and support food security in their household. Examining themes among rural households nationally with a low-income is an important step to develop and foster actions that can address food insecurity, which will contribute to addressing existing dietary and health disparities.

## 2. Materials and Methods

### 2.1. Study Design

A qualitative study design was applied to understand the strategies that households with a low-income in rural areas apply to support food security. Semi-structured interviews were conducted by female co-authors with credentials with low-income primary grocery shoppers from the households in six rural counties across Arkansas, Montana, North Carolina, Oregon, Texas, and West Virginia. Counties were first identified to participate based upon existing relationships that the researchers held within their home state and rurality as determined by a code of greater than 3 on the Rural-Urban Continuum Code (RUCC) [36]. Next, co-authors examined available data to reach consensus about the counties engaged in the study and identified variables that would ensure diverse representation across communities. Ultimately, counties were selected based upon the following criteria: poverty (federal poverty line based upon income and household size) or persistent poverty (20% or more of residents living in poverty since 1980) [37], a child food insecurity rate above 20% [38], racial/ethnic representation among African American, Hispanic/Latinx, or Native American within a majority of the counties [39], and a diverse political representation (Table 1) [40].

This study was approved by North Carolina (NC) State’s Institutional Review Board (IRB), Study #16-654. Affiliated universities deferred to the NC State IRB as the IRB of record, with the following exceptions: Salish Kootenai College’s Institutional Review Board approved this study, IRB Protocol #2019_5 and University of Arkansas for Medical Sciences, IRB Protocol #229190. 

### 2.2. Recruitment, Sample, and Setting

Researchers asked community partners and organizations to distribute flyers to recruit a convenience sample in each state during early 2019. Flyers were placed in community settings and distributed to potentially eligible individuals in those locations. Researchers desired to recruit approximately 25 participants in each state. Potential participants did not have a prior relationship with the research team, contacted the research team to indicate interest, and were screened via a basic demographic survey (i.e., sex, age, race, ethnicity, education, household composition, food security, primary food shopper status). Potential participants were eligible to participate if they identified as the primary food shopper in the household, were the caregiver for at least one child under the age of 18 in their household and responded affirmatively to at least one of the two-item food insecurity screening questions [41]. Of those who contacted the research team, 9 were ineligible. A total of 153 were eligible to participate. Interviews were conducted in private spaces within community settings that were convenient to the participant and the researcher. Co-authors discussed data saturation within each state and across the sample as interviews were occurring and when interviews were complete. Consensus occurred at the end of the interviewing stage that data saturation was reached.

### 2.3. Procedures

The semi-structured interview questions with probes were developed by the research team after reviewing the literature about rural food security and reaching consensus about salient topics that impact food security in rural areas. The researcher informed the participant about the study goals as a part of the consenting process prior to the interview starting. To pilot test, the research team conducted initial interviews, discussed the semi-structured interview guide in depth, and determined no revisions were necessary.

This study focused on a portion of the semi-structured interview related to participants’ ideal meal and successes, challenges, and strategies in feeding their family (Table 2). Including the first question provided the opportunity to understand the types of foods and meals that participants aspired to acquire, prepare, and consume within their household. The “ideal meal” explains how households with a low-income in rural areas aspire to compose meals, despite existing constraints when choosing foods. The remaining questions probed about strategies to acquire food during times of financial strain. 

After the interview, participants received $25 gift card or cash for their participation. Each participant provided written informed consent and participated in 1 interview. Each interview was conducted by a trained qualitative researcher co-author during 2019, lasted approximately 60 min, was audio-recorded. Those collecting data were trained to implement the semi-structured interview guide and take field notes. Interviews were audio-recorded and transcribed verbatim by a professional transcription company. A subset of interviews conducted in Spanish were translated after transcription. Transcripts were deidentified, examined for accuracy to prepare for qualitative analysis, and were not returned to participants for comment.

### 2.4. Data Analysis

Content analysis was applied throughout the coding process. Interviews were analyzed using the qualitative software program Atlas.ti version 7.0 (Scientific Software Development Gmbh, Berlin, Germany) [42,43,44]. Directed content analysis, which relies on an a priori coding scheme, was applied for the first interview question regarding participants’ ideal meal by study co-authors. Each food mentioned by a participant was coded by two researchers into the following major food groups according to the DGA: fruits, vegetables, grains/carbohydrates, discretionary calories, beans/peas/lentils, animal-based protein, and dairy. Foods mentioned were only placed in one category (e.g., beans were only placed in beans/peas/lentils and not in vegetables). If a participant mentioned a food more than once, the food was not double coded. The United States Department of Agriculture’s Food Patterns Equivalents Database (FPED) was used to confirm the food grouping for each food. For the remainder of the questions, a conventional content analysis approach was applied. First, each question was assigned to two researchers to independently code across all transcripts. Researchers created a codebook with codes that were derived from the responses to each question. The research team applied the codebook to four transcripts and updated the codes in an iterative process. Operational definitions were applied to the final codes. Researchers were trained to apply the final codebook to the by discussing each of the codes with examples. The final codebook was applied to all interviews. Coding discrepancies were resolved among the coders. When discrepancies arose, co-authors working in the state where the transcript originated were asked to weigh in to help resolve. Codes were collapsed into categories by consensus among researchers. Two themes emerged. Results were then organized by 2 themes, 10 categories, and illustrative quotes in the codebook [45]. The qualitative analysis and results adhere to the COREQ (Consolidated Criteria for Reporting Qualitative Research) guidelines for rigorous and systematic reporting of qualitative research. Initial analyses were sent to community partners that helped with recruitment to check on the findings. Quantitative demographic data was analyzed using JMP (version 15).

### 2.5. Reflexivity

The research arose from a collaboration between researchers from the Nutrition and Obesity Policy Research and Evaluation Network (NOPREN) Rural Food Access Working Group and staff from Feeding America and Share Our Strength. Authors recruiting participants had long standing relationships with the rural communities involved and lived and worked in the state that the data was collected. Authors collecting and analyzing data were skilled in qualitative methods and ethics through graduate degree programs and experience.

## 3. Results

In total, 153 individuals participated in the interviews across the counties in 6 states (Table 3). Most participants (88.9%) identified as female, with a mean age of 38.2 years and had a mean of 2.4 children living in their household. The most common race and ethnicity reported was Black/African American (40.5%) followed by White not Hispanic/Latino (25.5%), Hispanic/Latino (16.3%), and Native American (13.7%). Approximately 39.2% of participants completed high school or received a General Educational Development (GED) certificate. Most participants were either unemployed (33.9%) or employed outside of the home full-time (28.1%) and (62.1%) reported a monthly household income of less than $1980.

Nearly 70% of participants (67.3%) answered “sometimes true” to being worried that food would run out before they had money to buy more, while 23.5% reported that this statement was “often true”. The majority (64.1%) of participants answered, “sometimes true” when asked “within the past 12 months the food we bought just didn’t last and we didn’t have money to get more” and 18.3% said this statement was “often true”. Participants reported enrollment in nutrition assistance programs, including Supplemental Nutrition Assistance Program (SNAP) (73.2%), free or reduced-price school lunch or breakfast (69.9%), free groceries or meals (32.7%), and Special Supplemental Nutrition Program for Women, Infants, and Children WIC) (32.7%). 

### 3.1. Ideal Meal

**Figure 1:**
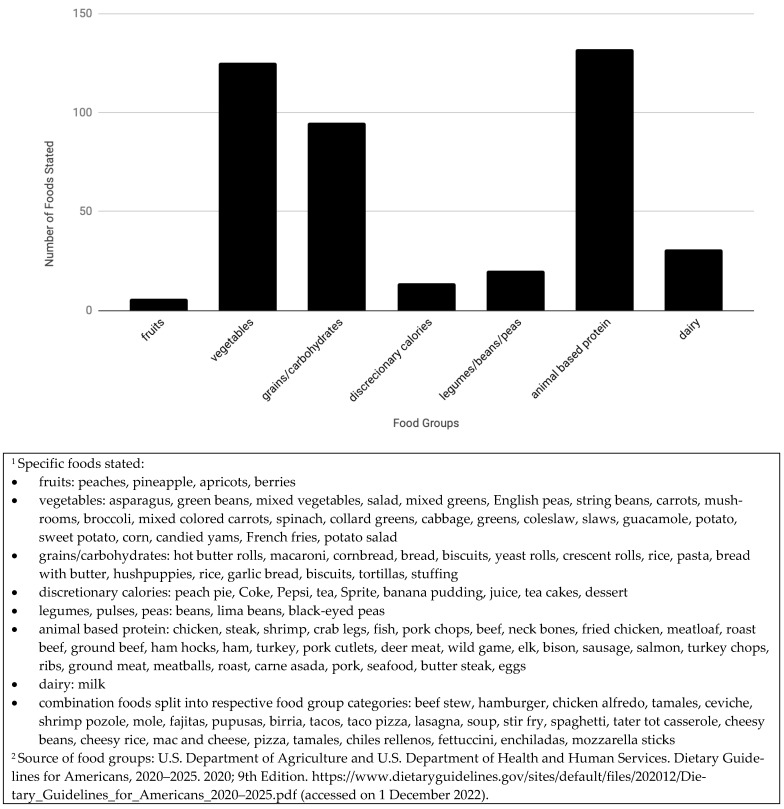
Ideal Meal Responses by Households with a Low-income in the Rural United States, 2019 (*n* = 132).

Overall, when asked “Tell me what your ideal meal is?” 132 participants responded to this question, 13 participants did not answer the question with enough detail to analyze the dietary components of the ideal meal, and 8 interviewers omitted the question due to the semi-structured interview nature of the study. Across all study sites, animal-based protein, grains, and vegetables were mentioned frequently. Nearly all participants included one or several animal-based proteins, or a combination food, such as pizza, that could include meat. Sixteen different types of grains were described, all of which could be refined or whole. Twenty four types of vegetables were described in participants’ ideal meals, including six starchy vegetables. Fruits were mentioned in 6 interviews and across only 3 states. Dairy products were referenced mostly as ingredients to combination foods such as macaroni and cheese. Legumes/beans/peas were mentioned in combination foods (i.e., entree dishes) or as a side. A small number of participants mentioned discretionary calories in desserts. As meal combinations, responses varied from very specific and culturally rooted “chili rellenos” to generalized responses like “a meat and two sides”. See Figure 1.

### 3.2. The Experience of Food Insecurity in Rural Areas

Two main themes arose from the analysis of interviews regarding the experience of households with a low-income and food insecurity in rural areas: (1) Struggles to Secure Food and (2) Coping Mechanisms to Secure Food. Categories for the Struggles to Secure Food theme included: Affordability, Adequacy, Accommodation, Appetite, and Time. Categories for the Coping Mechanisms to Secure Food theme included: Food Source Coordinating, Food Resource Management, Reduced Quality, Rationing for Food, and Exceptional Desperation. Table 4 describes each of the two major themes, categories, definitions, and representative quotes.

### 3.3. Struggles to Secure Food

The Struggles to Secure Food theme describe barriers to feeding oneself and family members, with 5 associated categories.

#### 3.3.1. Affordability

Affordability was commonly described in participant’s struggles to secure food. The cost of food was often too high, causing barriers to buy enough food or healthy food options. The inability to pay for food was a result of low-paying jobs or no jobs available in the area, financial insecurity due to unemployment or seasonal work, not having enough SNAP benefits to cover food costs, needing to use money for household or medical bills instead of food.

#### 3.3.2. Adequacy

Many participants reported struggles with running out of specific foods or lacking enough quality food, as they defined it. Across all states, participants described the struggle to maintain enough food in the house to feed their families. They expressed a desire for fresh and healthy food for their families spanning across different types of food groups. Participants knew that certain food groups, such as fruits and vegetables, were not as abundant to their families as necessary for good health. They described meals as inadequate when they could not achieve balance, typically with animal-based protein, grains, and vegetables. Some explained how higher-quality foods, such as fruits and vegetables, were sparse in their kitchens, and how, due to varying factors, healthy foods were lacking in their family’s diets while less expensive, more processed foods are purchased more often even though they realized they are not nutritionally adequate.

The struggle to secure adequate protein in participant’s diet was frequently mentioned. Protein was consistently indicated through the specific desire for enough meat or higher quality meat in a family’s diet. Some participants described how although meat is often one of the more expensive items they buy, it is the quickest to run out. Protein was indicated as important to some participants to meet their family’s appetite needs, keep family members full for longer, and create their ideal “complete” meals.

#### 3.3.3. Accommodation

In all states, participants described how working around picky eaters or differences in food preferences could be a struggle. A common issue described by participants was how to get children to eat fewer unhealthy, processed foods and more vegetables, when available. Some participants described the struggle of buying certain foods or having to prepare them in different ways for children, creating a need for extra time and financial burdens.

#### 3.3.4. Appetite

Participants described their struggles to satisfy family member’s seemingly insatiable hunger as a consistent challenge. Having enough food to satisfy young children, teenagers, and/or male partners was described in all states.

#### 3.3.5. Time

Participants explained that cooking healthy meals was difficult because it required a lot of time, compared to convenience meals which they perceived as easier and faster to prepare. Further, participants reported having to choose between taking the time to serve their families healthy food or preparing convenient, unhealthy meals, if they felt short on time, stressed, or tired. Struggles with time were associated with work and working late hours, time of transportation from retail food stores, schools, home, and planning times to cook and eat around various family members’ schedules, and exhaustion. 

#### 3.3.6. Coping Mechanisms

Five different categories of Coping Mechanisms were identified from the interviews within this theme. Each of the categories are strategies that participants employed to manage struggles to secure food. The data reflect that more than one coping mechanism was commonly used in a single household. Overall, the analysis demonstrated that the burden of the struggles to secure food and application of coping mechanisms were often placed on one family member (usually the mother or other female caregiver) who tended to be the person being interviewed. 

#### 3.3.7. Food Source Coordinating

Coordinated food resources is defined as synchronizing multiple systems and sources of food, including formal (e.g., federal food assistance) and informal systems (e.g., free meal or grocery programs), and alternative sources of food (e.g., donations from friends and family to meet food needs). 

Formal support systems were defined as federal and charitable food assistance programs such as SNAP and WIC, school meals, food pantries, food banks, and community meals. The use of formal support systems was the primary source of food support and was highly discussed regularly. Consistent codes for formal support systems correlate with the participation rates in SNAP, WIC, and school meal programs such as free and reduced-price breakfast and lunch. Participants rely on these programs to feed their families and incorporate them into their monthly budgets but mentioned that these programs do not completely meet their family’s food needs.

Informal support systems include receiving food from family and friends, borrowing money from family and friends. Informal support systems were described as a secondary source of support and not as highly used compared to formal support systems. 

Accessing alternative sources of food through activities like farming, gardening, hunting, fishing, canning, and other food preservation methods was used but reported less frequently than other methods to access food. These practices were described as important ways to honor generational ties as well as important food provisioning strategies. 

#### 3.3.8. Food Resource Management

A majority of participants demonstrated food resource management skills while shopping on a limited budget in order to secure food for their families. Participants coped with their struggles by buying only what was necessary, including choosing the same items every month to stay on budget, purchasing in bulk, selecting dented/damaged packages because they were lower cost, and choosing cheaper, generic brands. Participants traveled to multiple stores to use coupons and/or sales and compared unit prices when they shopped. 

#### 3.3.9. Reduced Quality

Reducing the quality of their meals or food as a coping mechanism was another way participants managed their limited food budgets and kept food on the table. Reduced quality was defined as improvising recipes or meals with foods that are not typically served together, eating and or buying food past the expiration date, choosing cheaper food options that are processed instead of fresh foods, and serving simple meals that have less variety. Participants consistently described buying cheaper food, letting milk or pantry items go past the expiration date, and making easy meals based on what was already in their kitchen.

#### 3.3.10. Rationing for Food

Participants described rationing food as eating less food during meals, reducing portion sizes, or using leftovers to stretch food so it would last. Participants explained that rationing food through leftovers ensured that food was not wasted and managed several struggles including limited time for food preparation. 

#### 3.3.11. Exceptional Desperation

Participants described exceptional desperation as experiences to feed themselves or their families that might be considered extreme in comparison to typical ways that individuals normally secure food. These coping strategies included adults skipping meals to prioritize children, selling or pawning items, stealing food or other items, avoiding wasting food scraps even when unappetizing, watering down food and beverages, and asking for discounts in stores. The data reflected the desire by individuals to ensure that their children were healthy. The most common practices were prioritizing the amount of food given to children over adults or ensuring that children, in comparison to adults, received higher quality food. As a last resort, participants watered down food and beverages for children to stretch its use.

## 4. Discussion

This study explored the ideal meals, struggles, and coping mechanisms of households with a low-income to obtain a desirable and nutritious meal when confronting food insecurity in six states of the rural U.S. Although geographic, cultural, and food system factors differed across the locations, the analysis demonstrated a common set of challenges and strategies that participants shared to secure food for their families.

### 4.1. Ideal Meal

Participants identified ideal meals that incorporated the food they preferred and could obtain, with consideration for nutrition. Participants’ ideal meals were somewhat aligned with the DGAs, prepared at home from scratch, and consisted of animal proteins, grains, and vegetables, which frequently resulted in a “meat and potatoes” pairing. Fruits and dairy products were mentioned infrequently, likely due to the expense of these food groups.

While the ideal meals described by participants did not completely match the DGAs, participants consistently described a desire to feed their families healthy foods that would stave off hunger. Ideal meals were likely influenced by societal norms, which are partially informed by expert dietary guidance over decades to make the “right choices” for a balanced meal [46]. However, the analysis demonstrated that participants across the six states struggled to provide their ideal meals due to adequacy, appetite, accommodation, time, and affordability. Further, even with the use of coping mechanisms, including rationing for food, exceptional desperation, food-management skills, reduced quality, and coordinating, providing their ideal meal was difficult.

### 4.2. Struggles to Secure Food

The most significant struggles to secure food were defined as affordability, adequacy, and accommodation. Overall, this research confirmed the findings of other studies, indicating that participants felt a daily sense of anxiety over the relentless inability to feed their family meals that were filling, affordable, healthy, and accommodated the needs of everyone in the household [47]. Research suggests that the daily requirement to negotiate basic needs and trade-offs for food insecure individuals leads to toxic stress for both children and adults [48,49,50,51,52]. In this research, parents tried to buffer their children from these impacts, but studies show that children are often aware of food insecurity and adopt their own strategies to mitigate hunger for themselves and other family members, compounding family stress [53].

Affordability was mentioned frequently as a struggle to eat, despite most participants reported usage of formal support systems such as SNAP and WIC and informal support systems such as friends and family. This finding was not surprising as food insecurity is often driven by a lack of financial resources [4,5,6,54,55,56]. Particular to the rural context, participant’s reliance on their network for food or financial support was notable. In addition, unemployment and underemployment are consistent issues in rural communities, with some counties struggling with persistent and/or extreme poverty. Participants in this study described consistent struggles to afford healthier, fresh foods such as fruits, vegetables, and meats, especially because of other bills (e.g., housing, electricity).

In comparison to these findings depicting daily struggles, data from a subset of participants described enjoying desserts and other specialty foods, reminiscent of findings that portray how food is not solely for the purpose of nourishment, but also tied to comfort, taste, celebration, or joy [57]. This analysis also showed that shared, historical memories played a role in how participants defined their ideal meals. They shared how they preferred to eat foods that connected to their traditions, such as wild game, candied yams, pupusas, and ceviche. Having the resources to make home-cooked meals that were culturally rooted was important, but sometimes not possible due to expense or access to certain foods.

The issue of children being “picky eaters” showed up in the data as part of the struggles participants described. Analysis of participants’ responses described how difficult it was to manage dietary goals and financial struggles, while also accommodating children who expressed strong preferences. Many families struggled with getting their children to eat enough food or healthier meals. Families with a low-income often do not have the time or resources to cook specific meals for children, to experiment with different types of food, try foods multiple times, and have the added worry that if a child does not like a meal, it could be a waste of resources [54,55,56]. Participants in this study demonstrated additional obstacles for feeding children that are unique to food insecurity, along with the struggle of picky eaters [48,51,53,57,58,59,60].

While having enough time to shop and prepare food showed up in the results, the analysis did not find time to be an overarching factor in how participants struggled to be food secure. Financial struggles were the biggest challenge for participants due to underemployment or unemployment, as they balanced limited budgets, competing appetites, and the desire to provide healthy meals for their household.

### 4.3. Coping Mechanisms

Consistent with other literature on food insecurity, participants described several coping mechanisms they used to deal with the struggle to ensure adequate food. The analysis resulted in five coping mechanisms categorized as: food source coordinating, food resource management, reduced quality, rationing for food, and exceptional desperation. Further analysis demonstrated that it was common to find the use of multiple coping mechanisms in a household.

This study described several coping mechanisms to prepare and provide meals for a family, including food resource management strategies like buying foods that were almost expired or damaged packaging and piecing together meals with whatever was on hand. Participants also reported provisioning or rationing food, using leftovers, and drawing on intergenerational homesteading practices [61]. Additionally, parents were eager to protect children from the negative physical and mental health outcomes of food insecurity and often prioritized children’s needs over their own. These coping mechanisms of exceptional desperation and accommodation reflect previous research, about how caregivers, despite utilizing all their resources, manage extraordinarily difficult circumstances and continue to struggle mightily to keep their families fed [48,53].

Amidst these financial struggles, participants worked hard to feed their families a balanced diet with the kinds of food their families liked to eat, generally described as a diet rich in animal proteins, grains, and vegetables. Participants knew that cheaper and heavily processed food were not as healthy for their families, but it was what they could afford. This situation likely drives the fact that individuals with low-income are more likely to consume ultra-processed food in the U.S. [62,63].

Most participants were enrolled in federal food assistance such as SNAP, WIC, or school meal programs. These federal nutrition programs are, by design, intended to be supplemental to support food security. It was clear that federal food assistance contributed to the participant’s food security but was not the entire solution to address all of a family’s food needs due to, in large part, affordability of adequate food. These findings are especially important to consider in context as rural households are often in communities with majority low-wage jobs, have higher rates of unemployment and underemployment, and have transportation barriers that limit access to food sources, increasing rural rates of food insecurity compared to urban counterparts [27,28,29,64]

Limitations to this research exist. Given that the data was collected from 25 to 28 individuals across 6 diverse geographic, economic, ethnic, racial, and politically leaning counties in 6 different states, the research findings are limited in generalizability and should be interpreted with caution. The participants were informed about the study goals as a part of the consenting process, which may bias response. This research examined rural experiences and would benefit from a comparative study with urban participants. A representative, quantitative study that examines these issues would enhance the application of results.

## 5. Conclusions

Despite various coping mechanisms, rural households with a low-income still struggle to feed their family desirable and nutritious food. This research describes how the eating patterns of families with a low-income are shaped by the rural settings in the U.S. Food experts, such as nutritionists, practitioners in the food system, researchers, health practitioners, may be able to use the research findings as a context to work in collaboration with families in rural areas with a low-income, partner with sectors that lie inside and outside the traditional strategies that address food security, lead to further research efforts, and support families’ efforts to be food secure.

## Figures and Tables

**Table 1:** Study County Characteristics of six rural counties across Arkansas, Montana, North Carolina, Oregon, Texas, and West Virginia (*n* = 6).

State	County	RUCC Code [36]	Persistent Poverty [37]	Child Food Insecurity Rate (2016) [38]	African American (%) [39]	Hispanic/Latino (%) [39]	Native American (%) [39]	House Democrats (%) [40]
Arkansas	Phillips	6	Yes	31.0%	63.0%	1.7%	0.0%	24%
Montana	Lake	6	No	21.2%	0.8%	4.1%	24.4%	41%
North Carolina	Halifax	4	Yes	26.8%	54.1%	2.6%	3.4%	38%
Oregon	Jefferson	6	No	24.2%	1.2%	19.8%	16.2%	58%
West Virginia	Calhoun	8	Yes	23.4%	0.2%	1.1%	0.1%	36%
Texas	Grimes	6	No	27.5%	17.1%	23.0%	0.2%	37%

**Table 2:** Qualitative Interview Questions Asked to Participants in Rural Households with a Low-Income about Food Security.

(1) Tell me what your ideal meal is? (2) We know from talking with lots of other people that it’s really hard to feed a family. What do you think you are doing really well when it comes to feeding your family? (3) What do you struggle with when feeding your family? (4) In the past year, has your family struggled to make ends meet? How did you get food during that time? Probe: How have these changes impacted the way you all eat? Probe: How have these changes impact the quality of meals you eat? Probe: What are some ways you tried to stretch your food dollars?

**Table 3:** Study Participant Demographics.

Characteristics	Total Sample
Mean age (*n* = 151)	37.9 ± 12.2
Mean number of adults in household (*n* = 152)	1.76 ± 0.9
Mean number of children in household (*n* = 151)	2.4 ± 1.4
Race/Ethnicity (*n* = 152)	
Black/African American	62 (40.8%)
Hispanic/Latino	25 (16.5%)
Native American	21 (13.8%)
White	39 (25.7%)
Other	3 (2.0%)
Prefer not to answer	2 (1.3%)
Education level (*n* = 153)	
<8th Grade	10 (6.5%)
Some high school	22 (14.4%)
High school or GED	60 (39.2%)
Some college	41 (26.8%)
College degree	18 (11.8%)
>College	2 (1.3%)
Marital status (*n* = 153)	
Married/living with partner	62 (40.5%)
Never been married	53 (34.6%)
Divorced	19 (12.4%)
Separated	10 (6.5%)
Widowed	5 (3.3%)
Prefer not to answer	4 (2.6%)
100% Federal Poverty Level or Less (*n* = 153)	96 (63.0%)
Program Participation (*n* = 153)	
SNAP	112 (73.2%)
WIC	50 (43.7%)
Free or reduced-price lunch or breakfast	107 (70.0%)
Free groceries or meals	50 (43.7%)
FDPIR	2 (1.3%)
Medicaid	126 (58.3%)
TANF	16 (7.4%)
WorkFirst	8 (3.7%)
Unemployment benefits	4 (1.85%)
Social Security/Disability Benefits	27 (12.5%)
Other	47 (29.4%)
None	1 (0.7%)
Food Security Status ^1^ (*n* = 153)	
High or marginal food security	29 (19.0%)
Low food security	75 (49.0%)
Very low food security	49 (32.0%)

^1^ Calculated using the USDA’s 6-item food insecurity screener.

**Table 4:** Themes, Categories, and Quotes for Strategies Applied by Rural Households with a Low-income to Confront Food Security (*n* = 153).

**Struggles to Secure Food Categories**	**Definition of Categories**	**Illustrative Quotes**
Affordability	Ability to pay for food due to low-paying jobs, no jobs, or food prices; could include financial insecurity due to unemployment, seasonal work, not enough SNAP benefits, having to use money for household or medical bills instead of food	OR_02: Porque hay muy poco trabajo entonces ahí si tienes que estirar lo más que puedas porque la luz sube mucho, el gas sube mucho, todos los servicios de casa suben más y no hay suficientes fondos para alcanzar. Con suficiente comida tienes que limitarte porque regularmente aquí trabajamos en el campo y el esposo es el que más trabaja y en el invierno no tienen trabajo. Translation: Because there is little work, you have to stretch (food) as much as possible because the light (bill) has increased a lot, the gas (bill) has increased a lot, all of the services of a house increase more and there are not sufficient funds to cover all of the costs. When you have sufficient food you need to limit yourself because normally here we work in the fields and the husband is the one who works the most and in the winter they do not have work.
Adequacy	Having enough of specific foods or a lack of enough quality food. Fruits and vegetables and proteins (i.e., meat) were especially mentioned.	MT_07: Probably just having enough food. We don’t have a lot of the best foods, it’s really hard to buy fruit and vegetables and fresh produce all the time. You just can’t. And so pretty much everything’s processed. But we survive. But we eat a lot of meats in our house, so that’s probably—yeah.
Accommodation	Satisfying picky eaters or navigating differences in food preferences, especially vegetables	TX_18: They’re picky. Kids are picky. So, one might not like carrots, the other one might like carrots, so I still put vegetables in my food, and they still have to eat vegetables. If one doesn’t like the carrot, he might not eat them, but the other one might eat them.
Appetite	Satiating a family member’s (usually child or male partner) insatiable hunger	WV_23: I’ve got two teenagers that can really eat. I mean, they can go through food; that’s why I go to the store about every week. I can’t keep food in the house.
Time	Lacking required time to cook healthy meals drives providing convenience food rather than preparing meals	AR_05: It seemed like whenever me and him were both working, I was just so tired and exhausted because I was also in school, as well. So, it seems like we ate out more because I just didn’t feel like coming home and cooking a full course meal and stuff. So, I think that’s where it affected us the most. Like I said, instead of just going and buying something to cook, you kind of just go buy something that’s already cooked and that’s not good.
**Coping Mechanisms to Secure Food Categories**	**Definition of Categories**	**Illustrative Quote**
Food Source Coordinating	Drawing upon multiple support systems to obtain food, including informal, formal, and alternative systems for sources of food.	WV_14: Food pantries here at [local organization], SNAP, and if we’re running low on anything usually the family will help.
	Support systems defined as: Informal support system: receiving food from family and friends, borrowing money from family and friends	NC_11: The entire check, I had nothing left, so I had to ask a couple of friends for money, get my kids something to eat, some snacks for school.
	Formal support system: signing up for SNAP/WIC, school meals, utilizing food pantries and food banks, community meals	MT_22: I like to try to stay close to our native foods that our ancestors grew; a lot of berries, a lot of—roots and things and—and deer meat—game, wild game. I try to stick to that.
	Alternative sources of food: gardening, hunting, fishing, canning	TX_07: Well, if it wasn’t for SNAP, I couldn’t feed ‘em [my family].
Food Resource Management	Exhibiting skill and desire to shop for and prepare healthy food, including buying only what is necessary, choosing the same items every month to stay on budget, going to multiple stores based on coupons and/or sales, buying in bulk, buying dented/damaged packages, choosing generic brands, comparing unit prices, cooking meals from scratch, or freezing and canning	NC_11: I would say giving them different options with food. A lot of people like to cook the same thing. If I have maybe a box of the instant noodles, like macaroni noodles and I have some vegetables in there, a can of vegetables or maybe some string beans or something, I’m going to try to make some type of pasta, soup or something. I love to try new things and that’s when you know you can really cook anyway when you ain’t got much to go off of.
Reduced Quality	Improvising recipes/meals with foods that are not typically served together, eating/buying food past the expiration date, choosing cheaper options that are processed instead of fresh foods, serving simple meals with less variety	TX_23: Yeah, sometimes the milk will probably be two or three days after. But, no more than three days after the date. Q: Okay. And then have you ever watered down food or drinks to make it last longer? TX_23: Yeah, some macaroni and cheese.
Rationing for Food	Eating less at meals, conservative portion sizes, stretching food to make efficient meals, using leftovers even when not a desirable combination	OR_08: We eat just a little piece each. I actually did barbeque yesterday night—yesterday afternoon and I did how I normally did it and a lot of it was left. So this morning my husband had leftovers and there’s still leftovers now so I think we’re gonna be eating that the rest of the day today.
Exceptional Desperation	Adults skipping meals for children, selling/pawning items, stealing food/items, avoiding wasting food scraps even when unappetizing, watering down food and drink, asking for discounts	NC_07: To be honest, I would steal sometimes, that’s all. I would go steal stuff and just to get food for my daughter. Q: And you never got caught? NC_07: No—well, one time, yeah, I did once before, I guess. Q: Did they—did you explain your—I’m curious, did you explain your situation? Did they let you go, and were they understanding? NC_07: No, well, they took me to jail. Q: Oh, they took you to jail? NC_07: I went to jail and I got probation for it. I was on probation for a year.

## Data Availability

Not applicable.
